# A rapid and robust method of identifying transformed *Arabidopsis thaliana *seedlings following floral dip transformation

**DOI:** 10.1186/1746-4811-2-19

**Published:** 2006-11-06

**Authors:** Samuel J Harrison, Ellie K Mott, Kate Parsley, Sue Aspinall, John C Gray, Amanda Cottage

**Affiliations:** 1 Department of Plant Sciences, University of Cambridge, Downing Street, Cambridge, CB2 3EA, UK

## Abstract

**Background:**

The floral dip method of transformation by immersion of inflorescences in a suspension of *Agrobacterium *is the method of choice for *Arabidopsis *transformation. The presence of a marker, usually antibiotic- or herbicide-resistance, allows identification of transformed seedlings from untransformed seedlings. Seedling selection is a lengthy process which does not always lead to easily identifiable transformants. Selection for kanamycin-, phosphinothricin- and hygromycin B-resistance commonly takes 7–10 d and high seedling density and fungal contamination may result in failure to recover transformants.

**Results:**

A method for identifying transformed seedlings in as little as 3.25 d has been developed. *Arabidopsis *T1 seeds obtained after floral dip transformation are plated on 1% agar containing MS medium and kanamycin, phosphinothricin or hygromycin B, as appropriate. After a 2-d stratification period, seeds are subjected to a regime of 4–6 h light, 48 h dark and 24 h light (3.25 d). Kanamycin-resistant and phosphinothricin-resistant seedlings are easily distinguished from non-resistant seedlings by green expanded cotyledons whereas non-resistant seedlings have pale unexpanded cotyledons. Seedlings grown on hygromycin B differ from those grown on kanamycin and phosphinothricin as both resistant and non-resistant seedlings are green. However, hygromycin B-resistant seedlings are easily identified as they have long hypocotyls (0.8–1.0 cm) whereas non-resistant seedlings have short hypocotyls (0.2–0.4 cm).

**Conclusion:**

The method presented here is an improvement on current selection methods as it allows quicker identification of transformed seedlings: transformed seedlings are easily discernable from non-transformants in as little as 3.25 d in comparison to the 7–10 d required for selection using current protocols.

## Background

*Arabidopsis *transformation mediated by vacuum infiltration of inflorescences with an *Agrobacterium *suspension was first introduced by Bechtold et al. [1]. This method has been widely used in preference to tissue culture techniques as it directly produces transformed seed and negates lengthy and complicated tissue culturing procedures. This transformation method was further modified by Clough and Bent [2] who demonstrated that the method was just as effective without vacuum infiltration. Immersion of *Arabidopsis *inflorescences in a suspension of *Agrobacterium*, with surfactant, was sufficient to generate transformed seed. This method remains the method of choice for *Arabidopsis *transformation. Selection of transformants from non-tranformants requires the presence of a marker, usually in the form of either antibiotic or herbicide resistance. Selection for resistance, for example to kanamycin, typically takes 7–10 d following germination [1,2].

Whereas advances in improving and simplifying the "floral dip" stage of the transformation have been developed, no significant advances in shortening or improving the selection process have been reported. Selection can be difficult; on germination, emerging cotyledons usually appear green, and transformed and non-transformed seedlings are frequently indistinguishable from each other. Over a period of 7–10 d, the non-transformants bleach and only the transformants remain green. During this lengthy period, fungal contamination may be a problem. Fungal contaminants may be present in seed stocks generated from floral dipping, because sucrose is used in the dipping medium and plants will have been, at least initially, kept in a warm damp environment providing ideal conditions for fungal growth. During seedling selection, fungal contaminants may deplete antibiotic or herbicide present in the selection medium, such that non-transformants are able to remain green. Crowding of seedlings on an agar plate may allow root growth above the medium and so result in a delay in bleaching of non-transformants due to decreased concentrations of selection agent. To address this problem, seedlings have been incubated in flasks of liquid growth medium on a rotary shaker instead of on agar-based medium [3]. This ensures dispersal of the floating seedlings across the medium surface and ensures that all are exposed to the antibiotic or herbicide. Seedlings are then sorted to identify those that are green. However, the procedure does not always work well and seedlings can be of poor quality, appearing flaccid and waterlogged; these seedlings seldom survive transplantation to soil.

We have developed a method of seedling selection that results in rapid, easy identification of transformants; the protocol presented works well for screening for resistance to kanamycin, phosphinothricin and hygromycin B.

## Results

**Figure 1:**
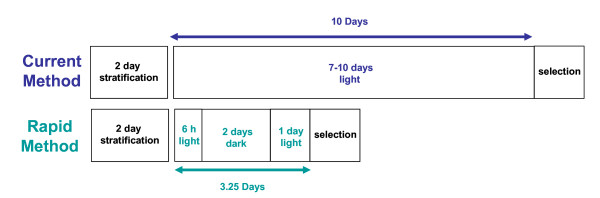
Time-line comparison of the current selection procedure and the rapid selection procedure.

Previously reported seed selection procedures requiring extended growth periods in the light result in seedlings with short hypocotyls [1,2]; this makes the transformants difficult to remove from agar plates and may result in damage to their cotyledons. To increase hypocotyl length and so facilitate easier seedling removal, we placed seedlings in the dark for 48 h before illumination. This produced a clear cut difference between resistant and non-resistant seedlings and allowed the development of a growth regime than can distinguish seedlings resistant to kanamycin, phosphinothricin or hygromycin B from non-resistant seedlings in 3.25 d, as opposed to the 7–10 d required in previously reported selection procedures (Figure 1). Following stratification for 2 d in the dark at 4°C, seeds were illuminated for 4–6 h to stimulate and synchronize germination and were then placed in the dark for 2 d where they continued to grow by hypocotyl extension. After a further 24-h light period, transformants were easily identified.

### Selection of kanamycin-resistant transformants

**Figure 2:**
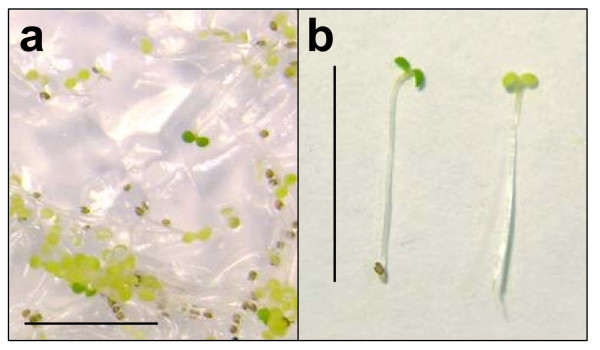
**Rapid selection of kanamycin-resistant *Arabidopsis thaliana *Col-0 seedlings. a. **Seedlings from *Arabidopsis thaliana *Col-0 T1 seed obtained from plants subjected to floral dip transformation using *Agrobacterium tumefaciens *strain GV3101 harbouring the binary plasmid pBINPLUS. Seeds were plated on 1% agar containing MS medium (including vitamins) and kanamycin at a concentration of 50 μg.ml^-1^. Following a stratification period of 2 d at 4°C in the dark, seedlings were incubated for 6 h at 22°C in white light, followed by incubation at 22°C in the dark for 2 d and continuous white light for a further 24 h. **b. **Kanamycin-resistant *Arabidopsis *seedling after rapid selection procedure (left); kanamycin-susceptible seedling after rapid selection procedure (right). Size bars 1 cm.

When wild-type and kanamycin-resistant seedlings growing on medium containing kanamycin (50 μg ml^-1^) were examined after the 2-d dark period, all cotyledons appeared yellow and closed, and hypocotyls were approximately 0.8–1.0 cm in length. A 24-h light period was sufficient for kanamycin-resistant transformants to accumulate chlorophyll and to continue to grow photoautotrophically. Transformants had green, open, expanded cotyledons (Figure 2). Non-transformants failed to accumulate chlorophyll; after 24 h in the light, they remained pale, with either closed or unexpanded cotyledons (Figure 2). This selection regime produced green transformants that were easily distinguished from yellow non-transformants on medium containing kanamycin.

To test the reliability of the selection protocol, it was applied to seeds derived from the transformation of *Arabidopsis *Col-0 with a construct containing *mGFP4 *[4] under the control of 707 bp of the 5' upstream sequence of the *CA1 *gene (At3g015000) to provide an easily identifiable reporter gene activity. The construct was inserted into the binary vector pBINPLUS containing the *nptII *selectable marker [5], and transferred into *Arabidopsis *by the floral dip method [2]. One hundred and eighteen kanamycin-resistant seedlings were identified by the rapid selection method, as outlined above, from about 3,200 seeds plated on sixteen 9-cm plates. All kanamycin-resistant seedlings were examined for *GFP *expression using fluorescence microscopy. One hundred and ten resistant seedlings (93%) showed clearly detectable GFP fluorescence. In three of the remainding eight kanamycin-resistant GFP-negative seedlings, the presence of *mGFP4 *was detected by PCR. Only one GFP-positive seedling was detected using fluorescence microscopy among ~800 kanamycin-susceptible seedlings visualised. This demonstrates a high degree of coincidence of kanamycin-resistant and *GFP*-expressing seedlings, and confirms the rapid selection method is comparable to traditional selection methods in the proportions of false positives and false negatives obtained.

A similar experiment was carried out with seeds derived from a floral-dip transformation with a pBINPLUS construct containing the *uidA *reporter gene [6] under the control of the *CA1 *promoter. Twenty kanamycin-resistant seedlings were selected by the rapid selection method from about 600 seeds plated on three 9-cm plates, and all kanamycin-resistant seedlings and about 200 kanamycin-sensitive seedlings were assayed histochemically for β-glucuronidase (GUS) activity. Only one kanamycin-resistant seedling had no detectable GUS activity, and all kanamycin-susceptible seedlings assayed were negative for GUS activity. This confirms that the rapid selection method successfully identifies transformants and the level of false negatives is very low. No false positives were ever observed when wild-type Col-0 seeds were subjected to the rapid selection procedure in the presence of kanamycin (data not shown).

We have confirmed the robustness of this rapid selection method with different *Arabidopsis *ecotypes, grown on different agar media and with illumination at different light intensities. The rapid selection method, as described in the protocol, has been used successfully to identify transformants of Col-0, Ws and Ler-0 (data not shown). The addition of vitamins to the minimal MS salts had no effect on the identification of kanamycin-resistant seedlings. However, the addition of 2% sucrose affected the efficiency of the selection method due to the altered morphology of the seedlings. Seedlings grown on 2% sucrose showed decreased hypocotyl growth, and a longer dark period (3–4 d) was required to enable clear identification of kanamycin-resistant seedlings. The success of the selection method was not affected by illumination at three different light intensities (80 μmol m^-2 ^s^-1^, 120 μmol m^-2 ^s^-1 ^and 200 μmol m^-2 ^s^-1^). No differences in the greening of the kanamycin-resistant or wild-type seedlings were observed with these intensities of white light, in growth cabinet or growth room environments.

### Selection of phosphinothricin-resistant seedlings

**Figure 3:**
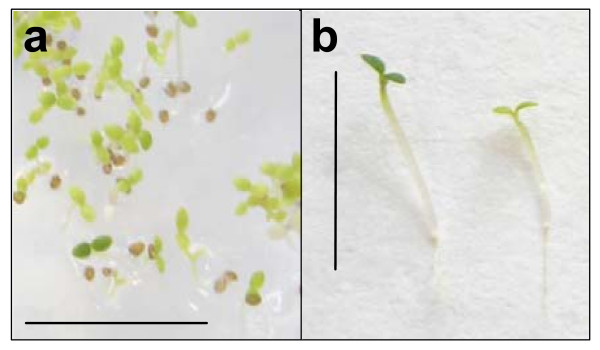
**Rapid selection of phosphinothricin-resistant *Arabidopsis thaliana *Ws seedlings. a. **Seedlings from *Arabidopsis thaliana *Ws T1 seed obtained from plants subjected to floral dip transformation using *Agrobacterium tumefaciens *strain GV3101 harbouring the binary plasmid pSKI015. Seeds were plated on 1% agar containing MS medium (including vitamins) and phosphinothricin at a concentration of 50 μM. Following a stratification period of 2 d at 4°C in the dark, seedlings were incubated for 6 h at 22°C in white light, followed by incubation at 22°C in the dark for 2 d and continuous white light for a further 24 h. **b. **Phosphinothricin-resistant *Arabidopsis *seedling after rapid selection procedure (left); phosphinothricin-susceptible seedling after rapid selection procedure (right). Size bars 1 cm.

The rapid selection method was also applied to a mixture of wild-type and phosphinothricin-resistant seedlings obtained following transformation with pSKI015 [7] which confers phosphinothricin resistance via the *bar *gene. In the presence of 50 μM phosphinothricin, dark-grown seedlings had extended hypocotyls (0.8–1.0 cm) and all seedlings had yellow, closed cotyledons. Following a 24-h light period phosphinothricin-resistant seedlings had green, open, expanded cotyledons whereas non-transformants had pale unexpanded cotyledons (Figure 3). To confirm the phenotypes of seedlings growing in the presence of phosphinothricin, wild-type controls were grown alongside previously characterised phosphinothricin-resistant lines under the conditions of the rapid selection method. All characterised phosphinothricin-resistant seedlings had green expanded cotyledons (~350 seedlings in total), whereas all wild-type controls had pale unexpanded cotyledons, with no exceptions (~400 seedlings in total). The presence of the *bar *gene was detected by PCR in all resistant seedlings sampled (2 seedlings from each of the control lines).

Several features of the selection procedure on phosphinothricin were examined in more detail, but only the addition of vitamins to the selection medium had any effect. The addition of vitamins slightly enhanced the greening of phosphinothricin-susceptible seedlings, but did not compromise the success of the selection method. The phosphinothricin-resistant seedlings were uniformly much greener than the resistant seedlings.

### Selection of hygromycin-resistant transformants

**Figure 4:**
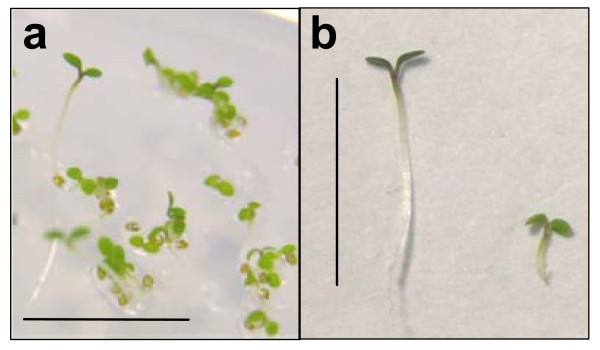
**Rapid selection of hygromycin B-resistant *Arabidopsis thaliana *Col-0 seedlings. a. **Seedlings from *Arabidopsis thaliana *Col-0 T1 seed obtained from plants subjected to floral dip transformation using *Agrobacterium tumefaciens *strain GV3101 harbouring the binary plasmid pBIG-HYG. Seeds were plated on 1% agar containing MS medium (including vitamins) and hygromycin B at a concentration of 15 μg.ml^-1^. Following a stratification period of 2 d at 4°C in the dark, seedlings were incubated for 6 h at 22°C in white light, followed by incubation at 22°C in the dark for 2 d and continuous white light for a further 24 h. **b. **Hygromycin B-resistant *Arabidopsis *seedling after rapid selection procedure (left); kanamycin-susceptible seedling after rapid selection procedure (right). Size bars 1 cm.

Seedlings grown in the presence of hygromycin B displayed a different morphology to those grown in the presence of kanamycin or phosphinothricin. After 2 d in the dark on medium containing hygromycin B (15 μg ml^-1^), hygromycin-resistant transformants had long hypocotyls of approximately 0.8–1.0 cm, whereas non-transformants had short hypocotyls (0.2–0.4 cm) (Figure 4). Additionally, in contrast to seedlings grown on kanamycin or phosphinothricin, all seedlings grown on medium containing hygromycin B were green after a 24-h light period. However, selection of hygromycin-resistant seedlings from non-transformants was easily achieved by selecting those with elongated hypocotyls.

To confirm the phenotypes of seedlings grown in the presence of hygromycin B, wild-type controls were grown alongside previously characterised hygromycin-resistant lines obtained following transformation with pBIG-HYG [8] which confers hygromycin B resistance via the *hpt *gene. The majority of characterised hygromycin-resistant seedlings had long hypocotyls following rapid selection. The presence of the transgene was detected in 16 randomly selected seedlings with this phenotype. Occasionally a few seedlings per plate had slightly shorter hypocotyls. For 5 independent lines containing the *hpt *gene the number of short seedlings were; 0/189, 4/194, 5/235, 0/177 and 0/201. However, the hypocotyls of these seedlings were not as short as those seen in wild-type Col-0 controls. In contrast, no hypocotyl elongation was seen in any wild-type Col-0 controls (~200 seedlings). The ability to distinguish hygromycin B-resistant and susceptible seedlings did not change following a further 2 d in the light.

## Discussion

We have developed a method that distinguishes kanamycin-, phosphinothricin- and hygromycin B-resistant seedlings from non-resistant seedlings in 3.25 d. This represents a considerable saving of time compared to current seedling selection methods which require a 7–10-d selection period. In addition, reducing the length of the selection period minimizes the risk of seedling loss by pathogen infection. The protocol produces easily identifiable resistant seedlings that are easily removed from selection plates, due to their long hypocotyls.

Kanamycin- or phosphinothricin-resistant seedlings can be distinguished from wild-type or non-transformant seedlings by the presence of chlorophyll in the expanded cotyledons. However, all seedlings grown in the presence of hygromycin B have green cotyledons, and the selection is based on hypocotyl length. Hygromycin-resistant seedlings have extended hypocotyls in the rapid selection method, whereas wild-type and non-transformant seedlings have short hypocotyls. The different effects of hygromycin B and kanamycin or phosphinothricin on hypocotyl elongation may be related to the target of these antibiotics. Hygromycin B inhibits cytosolic protein synthesis [9], whereas kanamycin and phosphinothricin inhibit plastid protein synthesis [10] and glutamine synthase activity [11,12], respectively. This suggests that cytosolic, but not plastid, protein synthesis is required for hypocotyl elongation in the dark. The length of hypocotyl extension in the dark is proportional to the amount of lipid reserve mobilized [13] and thus remaining reserves may influence the extent of chlorophyll synthesis on transfer of seedlings to the light. Wild-type or non-transformed seedlings grown on hygromycin B have short hypocotyls and are able to synthesize chlorophyll in the light, whereas wild-type or non-transformed seedlings grown on kanamycin and phosphinothricin have long hypocotyls and are unable to make chlorophyll. Lipid reserve mobilization is impaired in the presence of sucrose [14] and we observed that if sucrose was included in the selection medium a longer dark period (3–4 d) was required to enable clear identification of kanamycin- or phophinothricin-resistant transformants.

## Conclusion

We have developed a method that distinguishes kanamycin-, phosphinothricin- and hygromycin B-resistant seedlings from non-resistant seedlings in 3.25 d. Current seedling selection methods require a 7–10-d selection period. Reducing the selection period not only saves time but minimizes the risk of contamination. The protocol produces easily identifiable resistant seedlings that are easily removed from selection plates, due to their long hypocotyls. TThe he method as a laboratory-style protocol is available as a supplementary file see Additional file 1.

## Methods

### Plant material and transformation

Transformations of *Arabidopsis thaliana *were performed by the floral dip method [2] using *Agrobacterium tumefaciens *strain GV3101 [15]. The following binary plasmids were used: pBINPLUS [5] which confers kanamycin resistance via the *npt*II gene; pSKI015 [7] which confers phosphinothricin resistance via the *bar *gene; and pBIG-HYG [8] which confers hygromycin B resistance via the *hpt *gene. The reporter genes *uidA *with added intron [6] and *mGFP4 *[4] were inserted into the binary vector pBINPLUS under the control of 707 bp of the 5' upstream sequence of the carbonic anhydrase 1 (*CA1*) gene (At3g01500). The following non-transformed *Arabidopsis *seeds, obtained from the Nottingham *Arabidopsis *stock centre (NASC), were used for transformations and as controls; Col-0 (NASC N1092), Col-2 (NASC N907), Col-7 (NASC N3731), Ws (NASC N1601) and Ler-0 (NASC NW20). Previously characterised transgenic lines were used as positive controls; for phosphinothricin-resistance NASC accessions N21504, N21443, N21461, N21821, N21824 and N850573 were used, and for hygromycin-resistance 5 lines transformed with pBIG-HYG containing *mGFP4 *(K. Parsley unpublished data) were used.

### Plant selection

Seeds were surface sterilized by immersion in 70% (v/v) ethanol for 2 min, followed by immersion in 10% (v/v) sodium hypochlorite solution containing 8% available chlorine (Fisher Scientific, UK #S/5040/21) for 10 min. Seeds were then washed four times with sterile distilled water and sown onto 1% agar containing MS medium [16] and kanamycin monosulphate at a concentration of 50 μg ml^-1 ^(Melford Laboratories Ltd., Ipswich, UK #K0126), DL-phosphinothricin at a concentration of 50 μM (Melford Laboratories Ltd. #P01590250), or hygromycin B at a concentration of 15 μg ml^-1 ^(Melford Laboratories Ltd. #H0125). Excess surface liquid was drained from the plates. Seeds were then stratified for 2 d in the dark at 4°C. After stratification seeds were transferred to a growth chamber (Multitron, Infors UK, Reigate, UK) and incubated for 4–6 h at 22°C in continuous white light (120 μmol m^-2 ^s^1^) in order to stimulate germination. The plates were then wrapped in aluminium foil and incubated for 2 d at 22°C. The foil was removed and seedlings were incubated for 24–48 h at 22°C in continuous white light (120 μmol m^-2 ^s^-1^). The final 24–48 h light incubation need not be continuous; selection worked well when seedlings were placed in a 16 h light, 8 h dark regime although 24 h total light was required for optimum selection. This procedure has been carried out successfully using white light intensities of 80 μmol m^-2 ^s^-1^, 120 μmol m^-2 ^s^-1 ^and 200 μmol m^-2 ^s^-1 ^in both growth cabinet and growth room environments. The selection protocol also worked well on MS medium supplemented with additional vitamins (Melford Laboratories Ltd. #M0222) or 2% sucrose, but works optimally on MS medium alone.

### Expression studies using the uidA and GFP reporter genes

Kanamycin-resistant transformants identified using the rapid selection procedure were grown in a mixture (3:1 by volume) of standard potting compost (Levingtons M3) and vermiculite, under long-day conditions (16 h light, 8 h dark at 22°C). Staining for GUS activity was carried out on 1-cm leaf disc samples taken from 4-week-old plants, using 5-bromo-4-chloro-3-indoyl-β-D-glucuronide (X-Gluc) dissolved at a concentration of 1 mg.ml^-1 ^in 50 mM potassium phosphate buffer (pH 7.0) containing 0.05% Triton X-100 and 5 mM each potassium ferricyanide and potassium ferrocyanide [17]. Samples were incubated for 6 h at 37°C, cleared in 70% v/v ethanol and mounted in 50% v/v glycerol prior to examination. GUS activity in kanamycin-susceptible seedlings was detected by incubation of seedlings removed from the agar plates directly into the X-Gluc assay mixture, as described above.

The presence of GFP in 1-cm leaf disc samples from 4-week-old kanamycin-resistant plants was detected using a Nikon Optiphot-2 epifluorescence microscope with excitation and emission maxima of 395 and 450 nm, respectively. Kanamycin-susceptible seedlings were visualized on the agar plate for the presence of GFP using a Leica MZFLIII epifluorescence microsope.

### Transgene detection

DNA extractions and PCRs were performed using the REDExtract-N-Amp Plant PCR Kit (Sigma-Aldrich, #XNAP) in accordance with the manufacturer's instructions. The *bar *gene was detected in phophinothricin-resistant seedlings using primers *bar*F 5' TGCACCATCGTCAACCAC 3' and *bar*R 5' ACAGCGACCACGCTCTTG 3'. *mGFP4 *was detected in kanamycin- and hygromycin- resistant seedlings using primers *gfp*F 5' GGAGAAGAACTTTTCACTGG 3' and *gfp*R 5' GTAATCCCAGCAGCTGTTAC 3'. All PCR products were visualised on a 1.5% agarose gel containing ethidium bromide (1 μg.ml^-1^).

## Competing interests

The author(s) declare that they have no competing interests.

## Authors' contributions

SH was instrumental in developing the selection protocol. SH and EM were responsible for media preparation, seed sterilisation and plating. EM provided the seedling photographs. KP provided hygromycin-resistant lines, plasmid controls and primers. SA was responsible for plant growth, seed collection and ordering. AC prepared constructs and undertook all floral dipping. JG and AC both contributed to writing this manuscript. All authors have read and approved the final manuscript.

## Supplementary Material

Additional file 1 **A rapid and robust method of identifying transformed *Arabidopsis thaliana *seedlings following floral dip transformation**. The rapid selection method presented as a laboratory-style protocol. Click here for file
