# Supplementary material for: A rapid and robust method of identifying transformed Arabidopsis thaliana seedlings following floral dip transformation
Source: Plant Methods. 2006 Nov 6;2:19. doi: 10.1186/1746-4811-2-19 (PMC1636043; doi:10.1186/1746-4811-2-19)
Supplement: Additional file 1 — A rapid and robust method of identifying transformed Arabidopsis thaliana seedlings following floral dip transformation. The rapid selection method presented as a laboratory-style protocol. [file 1746-4811-2-19-S1.doc]

**A rapid and robust method of identifying transformed *Arabidopsis thaliana* seedlings following floral dip transformation**

1. In a 1.5 ml microfuge tube, surface sterilise 100 µl of seeds by adding 1 ml of 70% (v/v) ethanol and incubating for 2 min at room temperature. Remove the ethanol from the seeds and replace with 1 ml of 10% (v/v) sodium hypochlorite solution containing 8% available chlorine (Fisher Scientific, UK #S/5040/21). Incubate the seeds for 10 min at room temperature in the sodium hypochlorite solution. Remove the sodium hypochlorite from the seeds and replace with 1 ml of sterile water. Remove the water and replace with a fresh 1 ml of sterile water, repeat 3 times in order to fully wash the sodium hypochlorite from the seeds. Following washing, suspend seeds in a final volume of 500 µl of sterile water.
2. Pipette surface sterilized seeds onto 1% agar containing MS medium and kanamycin monosulphate at a concentration of 50 µg ml^-1^ (Melford Laboratories Ltd., Ipswich, UK #K0126), DL-phosphinothricin at a concentration of 50 µM (Melford Laboratories Ltd. #P01590250), or hygromycin B at a concentration of 15 µg ml^-1^ (Melford Laboratories Ltd. #H0125). Drain excess surface liquid from the plates.
3. Stratify seeds for 2 d in the dark at 4^o^C.
4. After stratification transfer seeds to a growth chamber and incubate for 4-6 h at 22^o^C in continuous white light (80-200 µmol m^-2^ s^1^) in order to stimulate germination.
5. Wrap plates in aluminium foil and incubate for 2 d at 22 ^o^C. Remove the foil and incubate seedlings for 24-48 h at 22^o^C in continuous white light (80-200 µmol m^-2^ s^-1^). Note: the final 24-48 h light incubation need not be continuous; selection works well when seedlings were placed in a 16-h light, 8-h dark regime, although 24-h total light was required for optimum selection.
6. Following the 24-48 h light period identify resistant/transformed seedlings from non-resistant/non-transformed seedlings as follows: kanamycin- resistant seedlings will have long hypocotyls and green cotyledons; whereas non-resistant seedlings will have long hypocotyls but pale cotyledons. Similarly, phosphinothricin-resistant seedlings will have long hypocotyls and green cotyledons, non-resistant seedlings will have long hypocotyls and pale cotyledons. In contrast, hygromycin B-resistant seedlings will have long hypocotyls and green cotyledons, whereas non-resistant seedlings will have short hypocotyls but will also have green cotyledons.
7. Following selection, carefully pull seedlings away from agar plates using forceps. Transplant seedlings into pots containing a mixture of standard potting compost (Levingtons M3) and vermiculite (Sinclair medium grade) 3:1 by volume. Place potted seedlings in a closed propagator, as initially seedlings need to be kept in a warm damp environment. Once seedlings are established remove the propagator lid and grow on to mature plants. Seedlings need not be removed from plates immediately following selection and can be left on plates for up to 1 week before transplantation.
